# The effect of post-COVID-19 ventilation measures on indoor air quality in primary schools

**DOI:** 10.1093/pnasnexus/pgad429

**Published:** 2023-12-20

**Authors:** Piet Eichholtz, Nils Kok, Xudong Sun

**Affiliations:** School of Business and Economics, Maastricht University, Tongersestraat 53, 6211 LM Maastricht, The Netherlands; School of Business and Economics, Maastricht University, Tongersestraat 53, 6211 LM Maastricht, The Netherlands; School of Business and Economics, Maastricht University, Tongersestraat 53, 6211 LM Maastricht, The Netherlands

**Keywords:** COVID-19, ventilation, indoor air quality, human behavior, cognition

## Abstract

The recent COVID-19 pandemic has made people acutely aware of the importance of indoor air quality (IAQ) and building ventilation systems, particularly in densely occupied places like offices and schools. As a result, governments and other public entities are increasingly investing in the installation, maintenance, and upgrades of ventilation systems in public buildings. However, little is known about the effect of building ventilation systems on actual IAQ and its impact on occupant behavior. This paper exploits exogenous closing and opening events of schools during the COVID-19 pandemic, combined with policy measures focusing on maximizing ventilation rates inside classrooms, to assess the effectiveness of building ventilation systems in primary schools. We use a unique sensor network implemented before the COVID-19 pandemic, consisting of measurement devices installed in 252 classrooms across 27 Dutch primary schools, continuously monitoring IAQ indicators such as CO_2_ levels and fine particle concentrations. Using high-frequency data from 2018 to 2022 school years, we compare the IAQ differences between natural and mechanical ventilation through a fixed-effect identification strategy. Our results show that mechanically ventilated classrooms perform better with respect to CO_2_ and fine particle levels. However, the post-COVID-19 ventilation measures implemented after school reopening had stronger effects on naturally ventilated (NV) classrooms, suggesting behavioral changes at the classroom level. We also investigate the longer term effects of these post-COVID-19 ventilation measures and show some evidence of decay in effectiveness, as well as a strong seasonal effect, particularly in NV classrooms, which seems the result of a trade-off between ventilation and thermal comfort.

**Significance Statement**

The COVID-19 pandemic underscored the importance of measuring and managing indoor air quality (IAQ) in buildings. This study assesses the effectiveness of building ventilation systems on indoor carbon dioxide and particle concentrations in Dutch primary schools from 2018 to 2022, and the impact of protocols aimed at changing ventilation behavior. Our findings contribute to understanding the impact of ventilation systems on IAQ and highlight the importance of human behavior in affecting real-world outcomes. The results can help inform future investments in school ventilation systems, ultimately enhancing the health and well-being, as well as the performance, of both students and staff.

## Introduction

The risk of airborne virus transmission in buildings has been of public concern throughout the COVID-19 pandemic (1), resulting in the temporary closing of both public and private buildings all over the world, including schools. This has led to detrimental effects on both learning outcomes and the mental health of students (2, 3). A less intrusive way to minimize the risk of airborne virus transmission in schools would be to substantially increase classroom ventilation rates (4). To some extent, this can be achieved by increasing ventilation rates in mechanically ventilated (MV) buildings or by opening windows and doors in buildings that lack mechanical ventilation (5). Most of these measures were included in post-COVID opening requirements for public spaces, especially schools. For example, after reopening, Dutch primary schools were required to keep the windows open or to keep ventilation systems running at full speed (6). However, many schools, both in Europe and North America, remain inadequately ventilated due to aging facilities, low-quality ventilation systems, and delayed or neglected maintenance (4).

The importance of classroom ventilation—and room ventilation more broadly—is reinforced by recent studies that have addressed the relationship between indoor air quality (IAQ) and cognitive performance. These studies have documented that improvements in IAQ have a significant impact on the cognitive performance of test subjects in laboratory settings (7), on the quality of thinking in chess tournaments (8), and on the outcomes of various cognitive tests (9). These indoor studies complement earlier work on the relationship between outdoor air quality and health (10, 11).

Good ventilation depends on both the performance of ventilation systems *and* on human behavior, such as opening windows or doors or increasing the intensity of mechanical ventilation (12). Before COVID-19, primary school teachers likely made different trade-offs between IAQ and classroom temperature (13), and schools made different trade-offs between IAQ and the energy cost implications of ventilation intensity (12). To evaluate the effect of ventilation systems and human behavior on IAQ, this paper exploits exogenous closing and reopening events in schools during the recent COVID-19 pandemic, combined with a public policy protocol focused on maximizing ventilation rates inside classrooms after reopening schools. The actual effect of these post-COVID measures relies on both mechanical and behavioral interventions.

## Results

### Main effects

Table 1 shows changes in CO_2_ levels for two post-COVID reopening periods, when compared with CO_2_ levels prepandemic. After the first reopening, we observed a significant decrease, of 18.5%, in daily average indoor CO_2_ levels and a 22.4% decrease in daily peak CO_2_ levels, when compared with pre-COVID levels. After the second reopening, we observe a significant decrease of 16% in average indoor CO_2_ levels and an 18% decrease in average daily peak CO_2_ levels, again when compared with prepandemic levels.

**Table 1.** Estimation results: the effect of the COVID-19 reopening protocol.

	Log (Average CO_2_)		Log (Peak CO_2_)	
	(1)	(2)	(3)	(4)
Reopening period 1	−0.185***	−0.188***	−0.224***	−0.227***
	(0.011)	(0.011)	(0.013)	(0.013)
Reopening period 2		−0.160***		−0.180***
		(0.012)		(0.014)
Sensor-fixed effect	Yes	Yes	Yes	Yes
Daily temperature controlled	Yes	Yes	Yes	Yes
Occupant number controlled	Yes	Yes	Yes	Yes
Observations	56,211	104,041	56,211	104,041
*R* ^2^	0.580	0.536	0.593	0.549
Adjusted *R*^2^	0.578	0.535	0.592	0.548
Residual SE	0.206	0.213	0.245	0.257

The table shows changes in CO_2_ levels for both reopening periods, when compared with CO_2_ levels pre-COVID. The reported numbers should be interpreted as percentage effects. For example, after the first reopening, we observed a significant decrease of 18.5% in average indoor CO_2_ levels and a 22.4% decrease in daily peak CO_2_ levels, when compared with pre-COVID levels. SEs are in parentheses. ***P < 0.01. *P < 0.1. **P < 0.05.

We further analyze the differences between mechanical and natural ventilation using the model of Eq. (1) (see Materials and methods) and provide results in Table 2. Columns (1) and (3) of Table 2 show the effect of reopening period 1 only (from prepandemic until December 2020), and columns (2) and (4) show changes for both periods. Importantly, for naturally ventilated (NV) classrooms, we find a significant decrease, of about 29%, in the daily average CO_2_ level. For MV classrooms, that decrease is smaller, but still a sizeable 15%. For daily peak CO_2_ levels, the numbers are somewhat larger, but the pattern is similar: NV classrooms have much larger improvements in IAQ with the implementation of the ventilation protocol. After the second reopening, the average CO_2_ level decreased by about 23% in NV classrooms when compared with the pre-COVID situation. That magnitude is smaller than the decrease after the first reopening. We also still observe a significant difference in the degree of CO_2_ reduction between mechanical and natural ventilation: the classrooms with mechanical ventilation show a smaller reduction in average daily CO_2_ levels than the other classrooms—some 9%.

**Table 2.** Estimation results: protocol effects per ventilation type.

	log(Average CO_2_)		log(Peak CO_2_)	
	(1)	(2)	(3)	(4)
Reopening period 1	−0.291***	−0.290***	−0.333***	−0.332***
	(0.020)	(0.020)	(0.024)	(0.024)
MV × reopening period 1	0.139***	0.132***	0.144***	0.136***
	(0.023)	(0.023)	(0.028)	(0.028)
Reopening period 2		−0.231***		−0.243***
		(0.021)		(0.024)
MV × reopening period 2		0.091***		0.080***
		(0.025)		(0.029)
Sensor-fixed effect	Yes	Yes	Yes	Yes
Daily temperature controlled	Yes	Yes	Yes	Yes
Occupant number controlled	Yes	Yes	Yes	Yes
Observations	56,211	104,041	56,211	104,041
*R* ^2^	0.587	0.540	0.598	0.552
Adjusted *R*^2^	0.585	0.539	0.596	0.551
Residual SE	0.204	0.212	0.243	0.256

Columns (1) and (2) show reopening effects for daily average CO_2_ levels, and columns (3) and (4) show effects for daily peak CO_2_ levels. Columns (1) and (3) show the effects for reopening period 1 only, and columns (2) and (4) show changes for both reopening periods. We distinguish MV and NV classrooms by including the interaction between the reopening period dummy and the mechanical ventilation dummy. We control for sensor-fixed effects, as well as daily average temperatures and classroom occupant numbers. The reported numbers should be interpreted as percentage effects. For example, after the first reopening, we observed a significant decrease of 29.1% in average indoor CO_2_ levels in NV classrooms, and 15.2% (29.1–13.9%) in MV classrooms. SEs are in parentheses. ***P  *<* 0.01. *P  *<* 0.1. **P  *<* 0.05.

### Dynamic effects

Next, we examine the monthly changes in IAQ improvement after school reopening. Based on findings from recent studies, people seem to adapt their response to COVID-19 measures in their daily activities and lifestyle (14, 15), such as continuously maintaining ventilation, especially when COVID-19 keeps its salience through news coverage (16). This would imply that IAQ in classrooms continues to improve in the short term, as people adapt to government measures and the ongoing information flow about COVID-19.

However, a competing hypothesis is that people forget about the invisible effects of ventilation and the corresponding health risks, or that they feel safe because of vaccination. As a result, people may revert to their original behavior, no longer strictly following the ventilation protocol that would otherwise enhance IAQ. The second scenario seems more likely, especially given the overall trend of lower rates of severe illness and higher vaccination rates in the COVID-19 epidemic after the closing and subsequent reopening of schools.

We first examine the dynamics of the change in IAQ levels by including the number of weeks after reopening in the baseline regression model. Table 3 demonstrates (under the assumption of linear variation) a slight upward trend in CO_2_ levels. After the first reopening (on 2020 May 11) and before the second school closure (2020 December 16), the average and peak CO_2_ levels rose by an average of 0.05% per week. At this speed, the improvements in IAQ reported above would be reduced to 0 in approximately half a year. However, the difference between ventilation types is quite significant: MV classrooms have an average weekly increase in CO_2_ level of 0.4%, when compared with a 0.8% weekly increase for NV classrooms—it seems that teacher behavior is such that windows and doors are increasingly closed again, as time since the school closing progresses.

**Table 3.** Estimation results: dynamic effects of the COVID-19 reopening protocol.

	log(Average CO_2_)		log(Peak CO_2_)	
	(1)	(2)	(3)	(4)
Number of weeks after reopening (NW)	0.005***	0.008***	0.006***	0.010***
	(0.001)	(0.001)	(0.001)	(0.001)
NW × mechanical ventilation		−0.004***		−0.006***
		(0.001)		(0.002)
Sensor-fixed effect	Yes	Yes	Yes	Yes
Daily temperature controlled	Yes	Yes	Yes	Yes
Occupant number controlled	Yes	Yes	Yes	Yes
Observations	15,891	15,891	15,891	15,891
*R* ^2^	0.577	0.582	0.569	0.574
Adjusted *R*^2^	0.571	0.576	0.563	0.567
Residual SE	0.164	0.163	0.207	0.206

The table shows the dynamics in daily average CO_2_ levels (columns 1 and 2) and daily peak CO_2_ levels (columns 3 and 4) after the first school reopening. Columns (2) and (4) distinguish mechanical and NV classrooms by including the interaction between the week after the first reopening and the mechanical ventilation dummy. We control for sensor-fixed effects, as well as daily average temperatures and classroom occupant numbers. The reported numbers should be interpreted as percentage effects. For example, average CO_2_ levels increase by 0.5% per week after the first school reopening. SEs are in parentheses. ***P < 0.01. *P < 0.1. **P < 0.05.

**Fig. 1.**
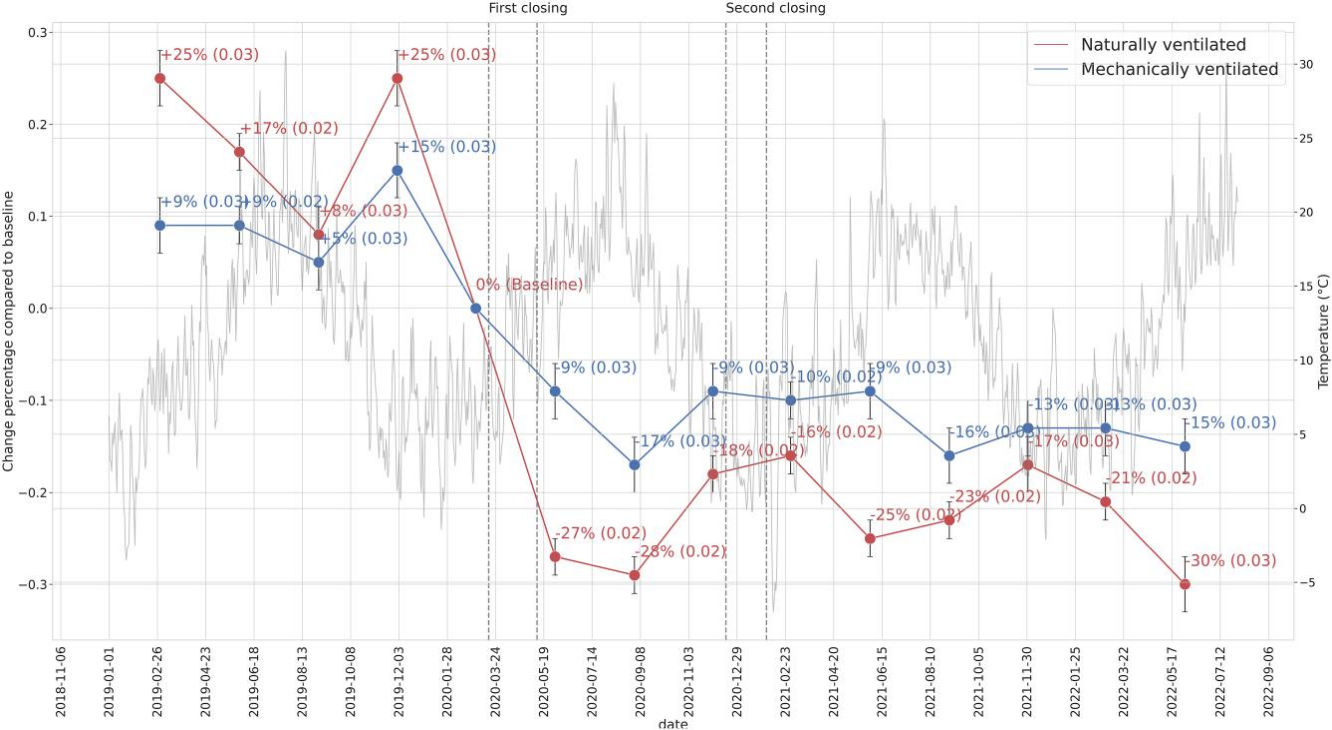
Dynamic effects. The graph depicts the change in CO_2_ levels relative to the average pre-COVID level, for consecutive observation periods of 90 days after the two school reopenings. The graph includes MV classrooms (the dark line started from +9%) and NV classrooms (the light line started from +25%). The *x*-axis shows the beginning and end dates for the 90-day observation periods, and the *y*-axis is the percentage change of daily (active hour) CO_2_ levels compared with the average level within a 90-day period before the first closing (set as 0). For example, the first point after the first closing on the upper line (−9%) indicates that, in 90 days after the first reopening (2020 December 5 to 2020 December 8), the average decrease in CO_2_ levels in MV classrooms is 9% (with an SE of 0.03) compared with the 90-day period before the first closing in MV classrooms. For reference, we added the daily temperature (light gray line) to show the seasonality of CO_2_ level changes. The daily temperature data (17) is provided by Royal Netherlands Meteorological Institute (KNMI, Koninklijk Nederlands Meteorologisch Instituut).

To further explore how the dynamic effect changes, we examine the change in CO_2_ levels over consecutive observation periods of 90 days after school reopening, as shown in Fig. 1. Here, we do not find a monotonic upward or downward trend in CO_2_ levels. Rather, two findings stand out. First, the reduction in CO_2_ level in MV rooms continues to be less than that in NV rooms. Second, Fig. 1 shows a clear seasonal effect, with CO_2_ levels generally higher in the colder seasons and much lower in the warmer months. While this seasonal effect is stronger in NV classrooms, it is also clearly visible in MV classrooms. This reflects the dynamic trade-off between ventilation quality and thermal comfort that teachers and their pupils face over the year.

### Fine particles

In the analysis above, we use CO_2_ as the main indicator of IAQ. However, fine particle levels are also an important aspect of the quality of indoor air, as these have been shown to directly affect human health and performance (18). Moreover, the observation of fine particles may be an indication of the presence and behavior of the aerosols that are material to COVID-19 and other virus contagion, as these are of comparable size (19).

Fine particle levels and CO_2_ levels may be substitutes or they may be complementary. Unlike CO_2_, which is mostly generated indoors (by human metabolism), particle pollutants are partially generated not only by indoor activities (e.g. dust) but also by outdoor pollutants (e.g. exhaust from automobiles). If the outdoor fine particle level is substantially higher than the indoor fine particle level, increasing ventilation will improve indoor CO_2_ levels but may worsen indoor fine particle levels, especially in a natural ventilation setting. Conversely, if the main source of fine particle pollution is indoors, increasing ventilation will improve both CO_2_ levels and fine particle pollution.

We again employ Eq. (1) to establish fine particle levels before, during, and after school closings, with appropriate controls, Table 4 shows the regression results: column (1) for average daily levels and column (2) for peak daily levels. We find that the trend of fine particle pollutants after reopening is like that of CO_2_, i.e. a significant overall decrease (13.9% for the first reopening and 18.9% for the second reopening, compared with the pre-COVID level), and the decrease of pollutants for classrooms with natural ventilation is larger than that for classrooms with mechanical ventilation. However, the effect difference between mechanical and natural ventilation is insignificant for most specifications reported in Table 4. This might be because a partial source of particle pollutants is outdoors. As windows are opened, outdoor pollution may offset some of the ventilation gains.

**Table 4.** Estimation results: ventilation measures and fine particles.

	log(Average PN1+)	log(Peak PN1+)
	(1)	(2)
Reopening period 1	−0.139***	−0.146***
	(0.029)	(0.026)
Mechanical ventilation × reopening period 1	0.075*	0.055
	(0.040)	(0.034)
Reopening period 2	−0.189***	−0.114***
	(0.043)	(0.036)
Mechanical ventilation × reopening period 2	0.056	0.007
	(0.056)	(0.045)
Sensor-fixed effect	Yes	Yes
Daily temperature controlled	Yes	Yes
Occupant number controlled	Yes	Yes
Observations	103,834	103,834
*R*2	0.521	0.366
Adjusted *R*^2^	0.520	0.364
Residual SE	0.569	0.558

This table shows estimation results for fine particle concentrations: daily average PN1+ levels in column (1) and daily average peak PN1+ levels in column (2). We distinguish mechanically and NV classrooms by including the interaction between the reopening period dummy and the mechanical ventilation dummy. We control for sensor-fixed effects, as well as daily average temperatures and classroom occupant numbers. The reported numbers should be interpreted as percentage effects. For example, after the first reopening, we observed a significant decrease of 13.9% in average daily PN1+ levels in NV classrooms and 6.4% (13.9–7.5%) in MV classrooms. SEs are in parentheses. ***P  *<* 0.01. *P  *<* 0.1. **P  *<* 0.05.

## Discussion

Air quality in buildings generally, and schools specifically, has become an important topic during the pandemic, given the airborne transmission of COVID-19 particles. More broadly, IAQ has been shown to affect human performance and learning outcomes (2, 7). This paper exploits exogenous closing and opening events in primary schools during the COVID-19 pandemic, combined with public policy protocol focusing on maximizing ventilation rates inside classrooms after reopening schools, to measure the effectiveness of natural and mechanical ventilation on CO_2_ levels and particulate matter (PM) concentrations. The actual effect of these post-COVID measures relies on both mechanical and behavioral interventions, and our interest is to understand the effectiveness of both interventions.

Importantly, our results demonstrate significantly enhanced IAQ after school reopening in both NV and MV classrooms, for CO_2_ levels as well as fine particle levels. Compared with MV classrooms, we document that NV classrooms improved more strongly. After the first school reopening, in May 2020, we observed a 29% drop in CO_2_ levels and a 14.6% drop in fine particle levels in NV classrooms. This compares to a decrease of 15.2% (CO_2_) and 6.4% (fine particles) observed in classrooms that are MV, after controlling for classroom and time-fixed effects, and for time-varying covariates that may influence air quality classroom (e.g. the number of students).

Our findings have some important policy implications. The knowledge about airborne transmission of disease has raised awareness of IAQ as a key factor in preventing the spread of contagious diseases. To prevent forced school closings in the future, many countries have begun to plan substantial investment projects to increase ventilation, whether through the renovation or installation of HVAC (heating, ventilation, and air conditioning) systems, or the updating of building ventilation regulations. Due to the high density of children in classrooms and historically inadequate investment in the maintenance or installation of air treatment systems, schools are among the major targets in many nations’ building portfolios.

However, even after the worries about COVID-19 recede, the salience of IAQ for learning outcomes will remain, given the importance of CO_2_ in affecting learning outcomes, and ventilation in schools is likely to remain a key policy issue for the foreseeable future. Our findings imply not only that installing and improving the functioning of mechanical ventilation can significantly affect IAQ, but also that behavioral intervention can be a simple and cost-effective complement in schools, states, or nations where natural ventilation is still common.

## Materials and methods

### Sensor infrastructure

**Fig. 2.**
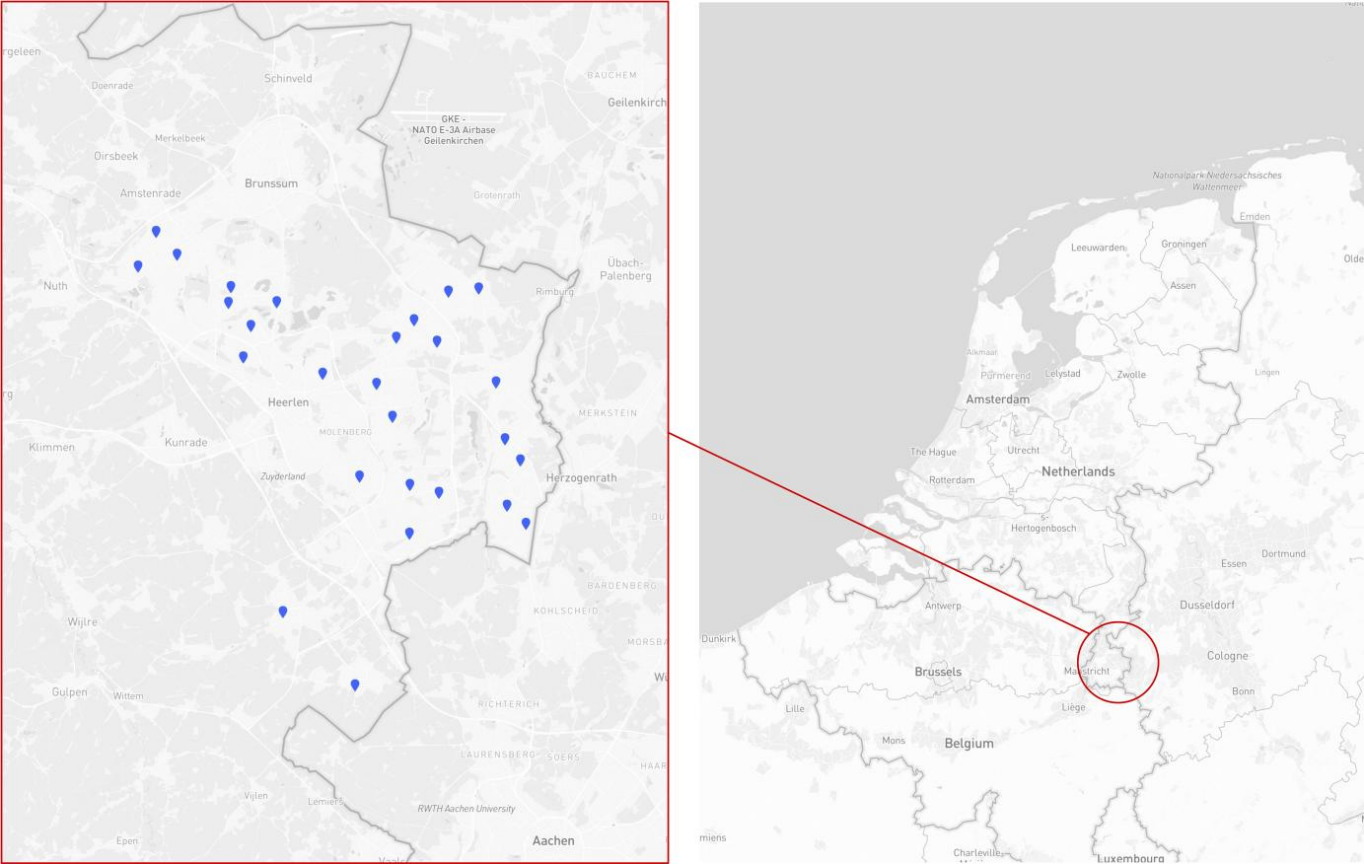
Locations of the primary school sample. The 27-school sample is randomly selected from the elementary schools managed by one school board in the south of Limburg province, The Netherlands.

In 2018, we created an infrastructure of IAQ sensors, using equipment from Aclima Inc., a San Francisco–based company. We installed sensors in 252 classrooms across 27 primary schools in the Limburg province, The Netherlands (2, 20). These schools have a combined overall enrollment of more than 5,000 students aged 5 to 12. Figure 2 shows the exact location of each school. These 27 schools represent a random sample of a larger school board with 47 schools under management, all in the same region. The school buildings in our sample are mostly built between 1980 and 2010, but we also observe a school built in 1932, and one built as recently as 2016. Seven of the 27 schools are NV, while 20 have mechanical ventilation. The information about the ventilation type (natural or mechanical ventilation) of each school building, as well as metadata about each school and classroom, is provided by Movare, the school board responsible for all schools used in this research.

Since we installed the sensor network in 2018, we have monitored a range of IAQ metrics, including CO_2_, fine particles, temperature, relative humidity, indoor light intensity, and sound. This study only considers the first two metrics: CO_2_, measured in parts per million (ppm), and fine particles, measured in particle (≥1 μm) numbers per liter of air (PN1+). We elected to focus on CO_2_ levels because of their relevance for learning outcomes (2), and fine particles because their size is comparable with the size of the aerosol droplets that are material in indoor COVID-19 contagion (19). For both metrics, we measure both the average concentration during the day, as well as their daily peak levels.

Before deployment, CO_2_ and PM sensors are calibrated at Aclima's facilities using reference-grade instruments, ensuring they meet precision, bias, and R-squared performance metrics. We refer to Table S2 for details on the sensor's performance and accuracy metrics. While raw data are gathered at intervals varying from 1 to 30 s, we utilize a smoothing procedure that aggregates the measurements to a 1-min resolution, using moving averages. A cloud-based tool was developed and deployed to monitor sensor status and performance, which involves routine network health assessments, sensor response checks, and data analysis to identify and investigate outlier sensors, thereby detecting any failures.

We complement the indoor sensor network with outdoor air quality sensors at four schools. This four-school subsample is geographically dispersed across the locations of our broader sample of 27 schools. The outdoor sensors are from PurpleAir and continuously monitor outdoor fine particle levels (outdoor CO_2_ levels are relatively constant over time and across locations).

### Study timeline

**Fig. 3.**
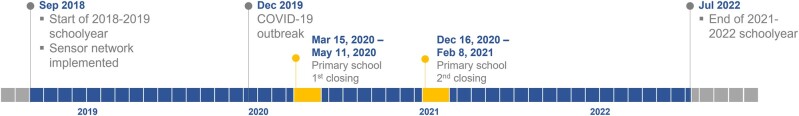
Timeline of COVID-19 school closings and reopening. The observation period covers time before, between, and after the school closings, allowing for a solid establishment of the baseline IAQ performance before the school closings, and for a sufficiently long observation of initial post-COVID performance, as well as the effects of a decay in ventilation intensity when COVID-19 concerns recede.

We used IAQ observations from September 2018 to July 2022, including data for four academic years. Figure 3 provides a timeline for our study, highlighting the school closings and reopening during the different stages of the COVID-19 pandemic. Before the first school closing period, school ventilation was not considered a (public) health priority for policymakers, school boards, teachers, and parents. That changed dramatically due to COVID-19, and after the first reopening, a national school ventilation protocol came into place that prescribed maximum mechanical and natural ventilation. As a result, school boards could no longer make a trade-off between electricity bills and ventilation outcomes, and ventilation systems were put to maximum levels. The same holds for teachers: outside temperature became less of a barrier to opening windows, as they were instructed to keep windows open continuously. In that sense, the COVID-19 school closings and reopenings amount to a natural experiment that allows us to investigate the effectiveness of (active) ventilation measures for IAQ.

The timeline also illustrates that we have a long observation period before, between, and after the school closings, allowing for a solid establishment of the baseline IAQ performance before school closings, and for a sufficiently long period of initial post-COVID performance, as well as the effects of a possible decay in ventilation intensity when COVID-19 concerns receded.

### Descriptive statistics

Table S1 shows average levels and standard deviations for the two IAQ variables of interest for this study: CO_2_ and fine particles. For both variables, we first show the average daily level, followed by the cross-classroom average of the daily peak levels, for the full sample period. For all measurements, we consider active hours only, i.e. the time during which a classroom is in actual use. We determine active hours using an algorithm that combines the levels of CO_2_ and sound in a classroom (2). The numbers reported in column (1) of Table S1 suggest that the IAQ in the average classroom, regarding the CO_2_ level, is narrowly compliant with Dutch regulations on indoor CO_2_ levels. For example, before the first COVID-19 lockdown, the CO_2_ level for the daily active hour average was about 1,000 ppm. Peak levels though, were at 1,476 ppm on average, which is clearly above regulatory levels (1,000 ppm for new construction and 1,200 ppm for existing buildings).

**Fig. 4.**
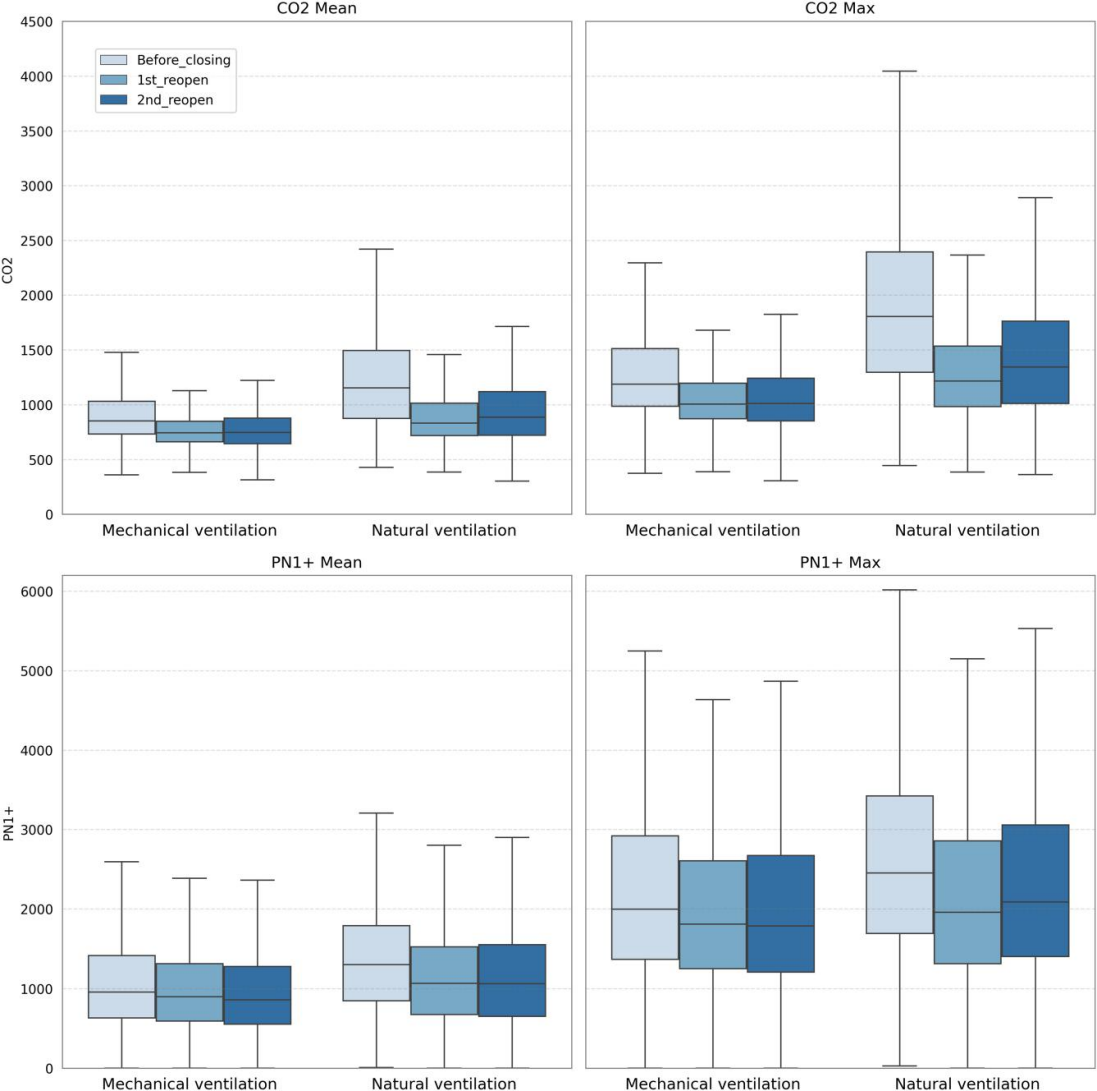
Daily average and daily peak CO_2_ and particle level comparison. The graphs depict CO_2_ and particle levels before the first school closing (2020 March 15), after the first reopening (2020 May 12 to 2020 December 15), and after the second reopening (2021 February 7 to 2022 July), both for schools with mechanical ventilation and for schools with natural ventilation. The left graph shows daily average levels and the right graph shows daily peak levels.

The averages reported in Table S1 hide a fairly large number of outliers and do not distinguish between MV and NV classrooms. Figure 4 provides the distribution of CO_2_ levels, showing substantial numbers of observations at very high levels during periods in which schools were open. We quite frequently observe CO_2_ levels between 1,000 and 3,000 ppm, levels that are known to have adverse consequences for cognition, decision-making, and learning outcomes (2). With respect to mechanical vs. natural ventilation, we document an advantage of mechanical ventilation when compared with natural ventilation, both in terms of daily average CO_2_ concentration and in terms of daily peak CO_2_ concentration. That difference holds both before the COVID-19 pandemic and after the reopening of schools. However, the difference between mechanical ventilation and natural ventilation is economically not very large and became even smaller after the schools reopened, e.g. an average of 911 vs. 1,212 ppm before COVID-19 and 783 vs. 918 ppm after school reopening.

Using data from a large-scale sensor network deployed in 252 classrooms across 27 primary schools, implemented before the start of the COVID-19 pandemic, to put these levels into perspective, existing studies (2, 7, 21) suggest that exposure to CO_2_ levels higher than 1,000 ppm could lead to reduced human performance, including learning, even after a few hours, and to health risks when the exposure is chronic. Some studies even recommend a lower threshold, at 800 ppm.

### Mathematical evaluation

To evaluate the efficacy of mechanical ventilation in enhancing indoor quality, and the effect of increased ventilation intensity after school reopening (both for mechanical and for natural ventilation), we use a fixed-effect approach comparing the IAQ in classrooms before and after school closings. We estimate the following empirical model:


(1) Yi,t=σn⋅Reopeni,t+δ⋅MV⋅Reopeni,t+β⋅Xi,t+μi+τt+εi,t

where *i* indicates the measured classrooms, and *t* indicates dates. Our outcome variable *Y_i, t_* includes the set of metrics measuring the IAQ in the classroom *i* at time *t*. We include metrics measuring the CO_2_ level (in ppm) and the particle level.

As we have two closing periods, the variable Reopen*_i,t_* contains two dummy variables indicating whether time *t* is during the first reopening (2020 May 11 to 2020 December 15) or the second reopening (after 2021 February 8). *σ* describes the average change in *Y_i,t_* after the school reopening. We also tested for potential interaction effects between reopening and mechanical ventilation. *δ* captures the marginal change in IAQ, after reopening, in MV classrooms when compared with NV classrooms. *β* describes the effect of individual time-varying control variables, *X_i,t_*, including the teacher and the number of students assigned to each classroom. The classroom-fixed effects *µ_i_* should reduce bias resulting from differences between the MV and NV classrooms. *τ_t_* is the date-fixed effect, which controls for seasonal influences (i.e. weather), among others. Finally, *ε_i,t_* is the error term, where the residual is grouped at the level of each classroom.

The fixed-effect model above captures the overall changes in IAQ outcomes before and after policy interventions, i.e. the difference between the situation before and after school reopening. However, as discussed in previous sections, the IAQ outcome depends on a combined effect of mechanical ventilation systems and human behavior. In this respect, we can reasonably assume that people's behavior in classrooms can change over time because of various factors, such as the weather and COVID-19 awareness. For example, teachers will not keep windows open for long during cold weather. In addition, when the number of new COVID-19 cases is high, teachers are presumably likely to follow COVID-19 guidelines more strictly, including the opening of windows.

To capture this dynamic effect, we modify the previous model using an event study method, estimating the effect of changes in ventilation intensity in each additional month after school reopening:


(2) $$\begin{array}{rl} Y_{i,t} & =\overset{N}{\underset{n=1}{\sum }}\;\sigma_{n}\cdot {\mathrm{Reopen}}_{i,t}^{\left\langle m\right\rangle }+\overset{N}{\underset{n=1}{\sum }}\;\delta_{n}\cdot MV\cdot {\mathrm{Reopen}}_{i,t}^{\left\langle m\right\rangle }+\beta \cdot X_{i,t}+\mu_{i}\\ & \;+\tau_{t}+\varepsilon_{i,t} \end{array}$$

In this model, *σ_n_* describes the average change in *Y_i,t_*, *n* periods after school reopening. For example, if we take 1 month as one period, *σ*_3_ describes the change in *Y_i,t_* 3 months after reopening. Similarly, *δ_n_* captures the additional changes for MV classrooms compared with NV classrooms *n* periods after school reopening. Like Eq. (1), the variable Reopen*_i,t_* contains two dummy variables indicating the first (2020 May 11 to 2020 December 15) or the second reopening (after 2021 February 8).

## Supplementary Material

pgad429_Supplementary_Data

## Data Availability

The original climate sensor data and control variables created for this study will be available in the Open Science Framework (OSF) repository named “The Effect of Post-COVID Ventilation Measures on Indoor Air Quality in Primary Schools” upon publication. The data can be accessed via DOI: 10.17605/OSF.IO/ZRFST. The code to reproduce the results of this article is publicly available at https://github.com/alex-x-sun/COVID-ventilation.

## References

[1] Erath BD, Ferro AR. 2022. Infectious disease transmission from bioaerosols. J Expo Sci Environ Epidemiol. 32(5):645–646. 36097166 10.1038/s41370-022-00476-z PMC9465135

[2] Placios J, Duran N, Kok N, Eichholtz P. 2022. Indoor air quality and learning: evidence from a large field study in primary schools. MIT Center for Real Estate Research Paper (22/13).

[3] Levinson M, Geller AC, Allen JG. 2021. Health equity, schooling hesitancy, and the social determinants of learning. Lancet Reg Health Am. 2:100032. 36588583 10.1016/j.lana.2021.100032 PMC9790768

[4] Fisk WJ . 2017. The ventilation problem in schools: literature review. Indoor Air. 27:1039–1051. 28683161 10.1111/ina.12403

[5] Iwashita G, Akasaka H. 1997. The effects of human behavior on natural ventilation rate and indoor air environment in summer—a field study in southern Japan. Energy Build. 25:195–205.

[6] National Institute for Public Health and the Environment . Ventilation, air purification and COVID-19. https://www.rivm.nl/en/coronavirus-covid-19/ventilation.

[7] Allen JG, et al 2016. Associations of cognitive function scores with carbon dioxide, ventilation, and volatile organic compound exposures in office workers: a controlled exposure study of green and conventional office environments. Environ Health Perspect. 124:805–812. 26502459 10.1289/ehp.1510037 PMC4892924

[8] Künn S, Palacios J, Pestel N. 2023. Indoor air quality and strategic decision making. Manage Sci. 69:5354–5377

[9] Zhang X, Wargocki P, Lian Z, Thyregod C. 2017. Effects of exposure to carbon dioxide and bioeffluents on perceived air quality, self-assessed acute health symptoms, and cognitive performance. Indoor Air. 27:47–64. 26825447 10.1111/ina.12284

[10] Heft-Neal S, Burney J, Bendavid E, Burke M. 2018. Robust relationship between air quality and infant mortality in Africa. Nature. 559:254–258. 29950722 10.1038/s41586-018-0263-3

[11] Lelieveld J, Evans JS, Fnais M, Giannadaki D, Pozzer A. 2015. The contribution of outdoor air pollution sources to premature mortality on a global scale. Nature. 525:367–371. 26381985 10.1038/nature15371

[12] Park J, Kim H. 2012. A field study of occupant behavior and energy consumption in apartments with mechanical ventilation. Energy Build. 50:19–25.

[13] Kim H, Hong T, Kim J. 2019. Automatic ventilation control algorithm considering the indoor environmental quality factors and occupant ventilation behavior using a logistic regression model. Build Environ. 153:46–59.

[14] Maltagliati S, et al 2021. Evolution of physical activity habits after a context change: the case of COVID-19 lockdown. Br J Health Psychol. 26:1135–1154. 33822454 10.1111/bjhp.12524 PMC8250330

[15] Husain W, Ashkanani F. 2020. Does COVID-19 change dietary habits and lifestyle behaviours in Kuwait: a community-based cross-sectional study. Environ Prevent Med. 25:1–13. 10.1186/s12199-020-00901-5 PMC7548533 33045996

[16] Reshetnikov V, et al 2021. Indoor environmental quality in dwellings and lifestyle behaviors during the COVID-19 pandemic: Russian perspective. Int J Environ Res Public Health. 18:5975. 34199589 10.3390/ijerph18115975 PMC8199671

[17] Koninklijk Nederlands Meteorologisch Instituut (KNMI) . 2023. Daily weather data in the Netherlands. [accessed 2023 October 25]. https://www.knmi.nl/nederland-nu/klimatologie/daggegevens.

[18] Copat C, et al 2020. The role of air pollution (PM and NO_2_) in COVID-19 spread and lethality: a systematic review. Environ Res. 191:110129. 32853663 10.1016/j.envres.2020.110129 PMC7444490

[19] Zhao L, Liu J. 2020. Operating behavior and corresponding performance of mechanical ventilation systems in Chinese residential buildings. Build Environ. 170:106600.

[20] Temprano JP, Eichholtz P, Willeboordse M, Kok N. 2020. Indoor environmental quality and learning outcomes: protocol on large-scale sensor deployment in schools. BMJ Open. 10(3):e031233. 10.1136/bmjopen-2019-031233 PMC7076238 32184302

[21] Jones AP . 1999. Indoor air quality and health. Atmos. Environ. 33:4535–4564.

