# Supplementary material for: The effect of post-COVID-19 ventilation measures on indoor air quality in primary schools
Source: PNAS Nexus. 2023 Dec 20;3(1):pgad429. doi: 10.1093/pnasnexus/pgad429 (PMC10748476; doi:10.1093/pnasnexus/pgad429)
Supplement: pgad429_Supplementary_Data [file pgad429_supplementary_data.docx]

**Appendix 1: Descriptive statistics**

The table shows daily average CO_2_ levels, daily peak CO_2_ levels, and daily average particle levels. The standard deviations are calculated across the classrooms and over time.

|  | Average | St. Dev. | Maximum |
| --- | --- | --- | --- |
| ***Period* 1: *before COVID-19*** |  |  |  |
| Average daily *CO*_2_ (*ppm*) | 1,000 | 392 | 4,690 |
| Peak daily *CO*_2_ (*ppm*) | 1,476 | 695 | 5,336 |
|  |  |  |  |
| Average daily particle (*PN*1^+^) | 1,156 | 783 | 38,919 |
| Peak daily particle (*PN*1^+^) | 2,660 | 3,558 | 227,541 |
| ***Period 2*: *1st reopening*** |  |  |  |
| Average daily *CO*_2_ (*ppm*) | 818 | 253 | 4,150 |
| Peak daily *CO*_2_ (*ppm*) | 1,164 | 456 | 5,177 |
|  |  |  |  |
| Average daily particle (*PN*1^+^) | 1,058 | 863 | 46,612 |
| Peak daily particle (*PN*1^+^) | 2,390 | 3,559 | 166,901 |
| ***Period 3*: *2nd reopening*** |  |  |  |
| Average daily *CO*_2_ (*ppm*) | 839 | 289 | 3,516 |
| Peak daily *CO*_2_ (*ppm*) | 1,216 | 540 | 5,061 |
|  |  |  |  |
| Average daily particle (*PN*1^+^) | 1,033 | 1,028 | 161,226 |
| Peak daily particle (*PN*1^+^) | 2,414 | 3,516 | 239,426 |

**Appendix 2: Sensor characteristics**

Technical specifications of the sensors, including sensing methods, accuracy, resolution, and sample frequency.

|  | Sensing method | Accuracy | Resolution | Sample frequency |
| --- | --- | --- | --- | --- |
| Carbon dioxide *CO*_2_ (*ppm*) | Non-dispersive infrared | 50 ppm + 3% | 10 ppm | 17 s |
| Coarse particles PM (count/L) | Optical, scattered light | 250 count/L+ 20% | 250 count/L | 30 s |
| Relative humidity (rh) | Complementary metal oxide semiconductor | 4% | 0.3% | 5 s |
| Light (lux) | Photodiode | 3 lux | NA | 1 s |
| Temperature (°C) | Solid state integrated circuit | 1°C | 0.2°C | 1s |
| Sound (dB) | Back electret | 5 dBA | 3 dBA | 1 s |

**Appendix 3: Model specification and sensitivity analysis**

To ensure the robustness of our models, we undertook a comprehensive sensitivity analysis. We first construct a correlation matrix to examine interrelationships among indoor air quality parameters. The figure below shows Pearson correlation coefficients for the daily averages of the set of indoor air quality parameters. For both mechanically ventilated (MV) and naturally ventilated (NV) classrooms, we observe a positive correlation between CO_2_ levels and particle levels. Notably, CO_2_ has a negative correlation with outdoor temperature and air pressure, though these correlations are not very strong.


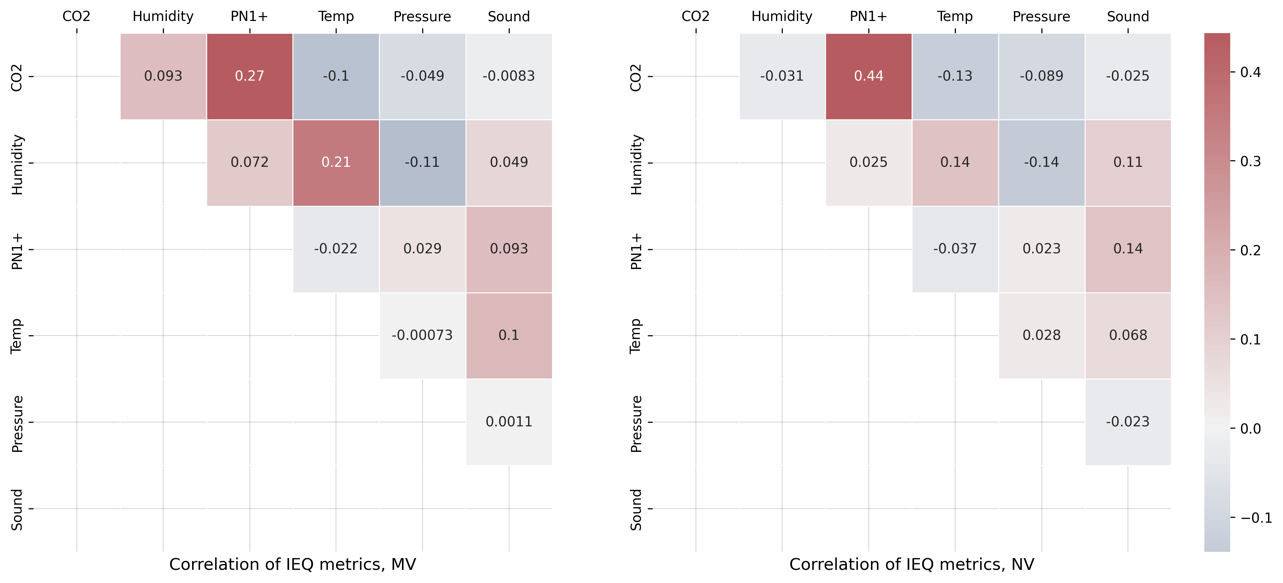


**Figure S1: Correlation matrix** The Pearson correlation coefficients for the daily averages of the set of indoor air quality parameters. For both mechanically ventilated (MV) and naturally ventilated (NV) classrooms

As a second sensitivity analysis, we estimate a large set of alternative models, including different definitions and combinations of control variables, to create a specification curve. The figure below plots coefficients from separate (standardized) estimates for four variables of interest: Reopening Period 1, MV*Reopening Period 1, Reopening Period 2, and MV*Reopening Period 2. Dots describe point estimates. Vertical lines indicate 95% confidence intervals. The consistency of the estimates across specifications supports the robustness of the findings and indicates that the results are not driven by a specific definition of exposure measurement.


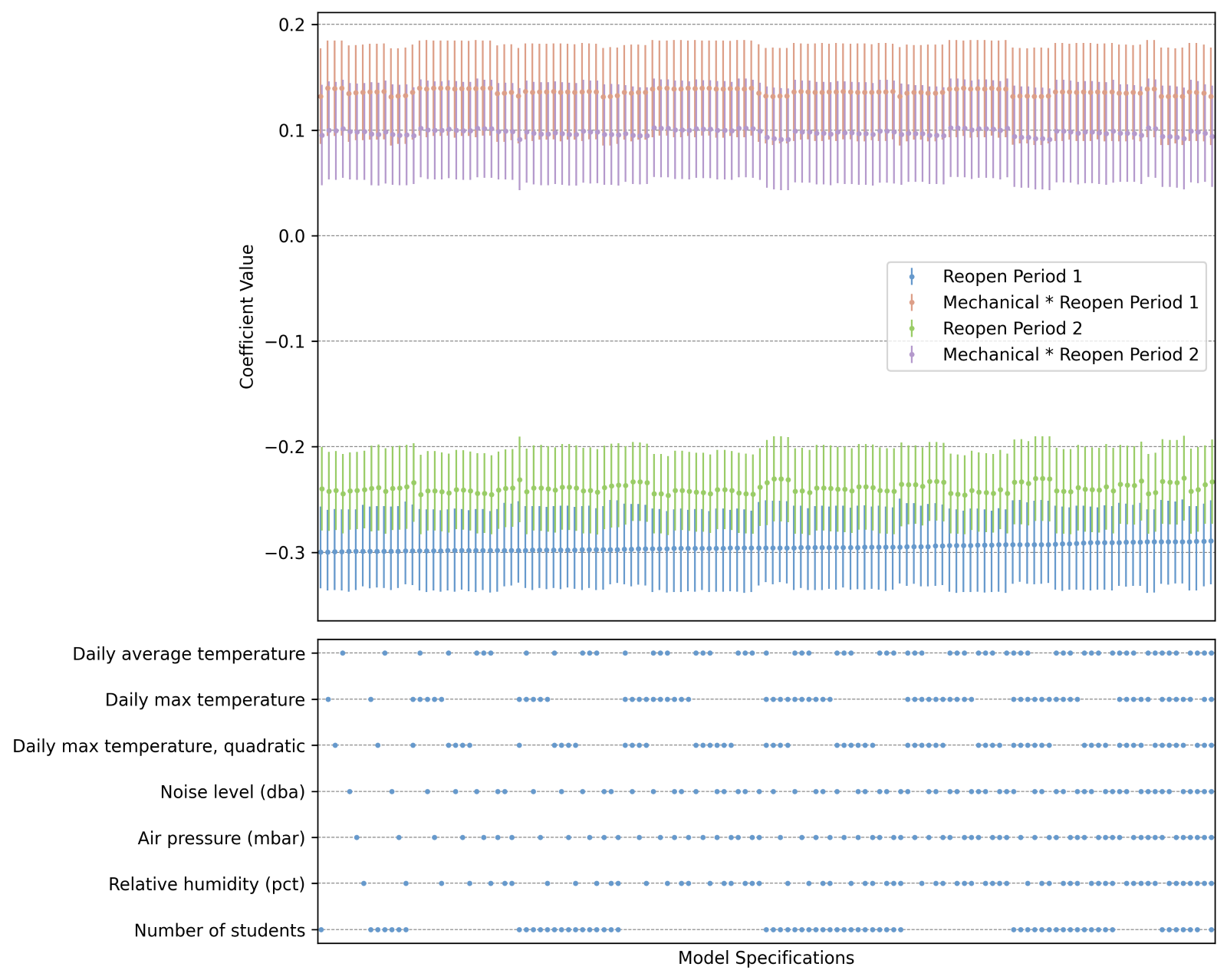


**Figure S2: Specification Curve** The figure plots coefficients from separate (standardized) estimates for four variables of interest: Reopening Period 1, MV*Reopening Period 1, Reopening Period 2, and MV*Reopening Period 2.
